# Global research landscape and emerging trends in Graves’ disease: A bibliometric analysis

**DOI:** 10.1097/MD.0000000000037963

**Published:** 2024-06-14

**Authors:** Yan Yang, Peijin Li, Chunjian Zhou, Feng Liu, Tao Liu, Qian Wang, Zhiguo Ding

**Affiliations:** a Department of Thyropathy, Dongzhimen Hospital, Beijing University of Chinese Medicine, Beijing, China; b Clinical Lab, Sunsimiao Hospital, Beijing University of Chinese Medicine, Tongchuan, Shanxi, China; c Department of Thyropathy, Sunsimiao Hospital, Beijing University of Chinese Medicine, Tongchuan, Shanxi, China; d The First People’s Hospital of Mudanjiang City, Mudanjiang, Heilongjiang Province, China; e Clinical lab, Tongchuan People’s Hospital, Tongchuan, Shanxi, China.

**Keywords:** bibliometrics, bibliometrix, China, CiteSpace, Graves’ disease, web of science

## Abstract

**Background:**

Graves’ disease is a prevalent thyroid disorder and is the primary cause of hyperthyroidism. Significant progress has been made in understanding the epidemiology, pathogenesis, diagnosis, treatment, and prognosis of this disease. However, bibliometric analyses on Graves’ disease are lacking. We aimed to comprehensively summarize the research, progression, and focal points of Graves’ disease through data mining and integrated analysis of the existing literature.

**Methods:**

We retrieved relevant literature on Graves’ disease from 2003 to 2023 from the Web of Science database. We performed bibliometric analysis using CiteSpace and the R package Bibliometrix.

**Results:**

We identified 10,901 publications from 132 countries, with a steady rise in the number of publications over the past 5 years. The US leads in publication volume, with the University of California System being the primary contributing institution. The journal Thyroid had the highest publication output, while the Journal of Clinical Endocrinology and Metabolism was the most frequently cited. These publications involved 2305 authors, with Antonelli Alessandro and Smith Terry being the most prolific. The most frequently cited articles were the “2016 American Thyroid Association guidelines for diagnosis and management of hyperthyroidism and other causes of thyrotoxicosis” and the “Thyroid Association/European Group on Graves’ orbitopathy guidelines for the management of Graves’ orbitopathy.” Analysis of the bursts of cited references, keywords, and their clustering revealed that research on Graves’ disease predominantly centers on clinical management, thyroid-stimulating hormone receptors, thyroid hormones, autoimmunity and inflammation, Graves’ ophthalmopathy, thyroid nodules, and thyroid cancer.

**Conclusion:**

This is the first comprehensive bibliometric study to summarize progress and trends in Graves’ disease research. These results highlight recent research hotspots and promising directions, thereby providing a valuable reference for other scholars.

## 1. Introduction

Graves’ disease (GD) is a thyroid-specific autoimmune disorder that constitutes a common thyroid ailment and is the primary cause of hyperthyroidism.^[1–4]^ It affects approximately 3% of women and 0.5% of men globally.^[5]^ The hallmark of this disease is the breakdown of immune tolerance to thyroid antigens, such as thyrotropin (TSH) receptor, thyroid peroxidase, and thyroglobulin, which possess unusual immunogenic properties, potentially leading to the collapse of immune tolerance and subsequent thyroid dysfunction.^[6–9]^

Currently, the research on the pathogenesis of GD is not very clear. Complex interactions between genetic susceptibility and environmental factors, such as viruses, radiation, drugs, and iodine, are considered major contributors to GD.^[10,11]^ These risk factors may lead to an imbalance of T lymphocyte subsets, resulting in increased differentiation of pro-inflammatory cells and decreased differentiation of anti-inflammatory cells. The immune imbalance in turn induces thyroid autoimmunity, resulting in thyroid cells presenting autoantigens directly to autoreactive CD4 + T helper cells. These cells are then activated, proliferate, and trigger the production of plasma cells that produce TSHR antibodies, leading to the occurrence of GD.^[2,3]^ The primary treatment for this condition comprises antithyroid medications. However, with the identification of risk factors and a clearer understanding of their pathogenesis, more promising therapeutic approaches (e.g., immunomodulation) are undergoing extensive clinical research.^[12]^ Over the past 2 decades, significant progress has been made in understanding the occurrence, development, and treatment of GD. The outcomes of prior studies form a foundation for future extensive in-depth research, aiding in the identification of hotspots and exploring new perspectives.

Bibliometric analysis is an emerging research method that involves the comprehensive analysis of various aspects of specific field-related literature, including publication quantity, journals, author information, citation frequency, and keywords. This approach aims to summarize past academic achievements and infer directions for future research and trends in a particular field.^[13]^ CiteSpace and the R package Bibliometrix are commonly used tools in bibliometric analysis. Besides extracting the aforementioned information, they facilitate visualization of the results,^[14,15]^ intuitively reveal the scientific development of cutting-edge knowledge, and help researchers grasp the current research status and trends in the field. Several bibliometric analyses have been conducted on Graves’ Ophthalmopathy, autoimmune thyroiditis, Hashimoto’ s thyroiditis, and thyroid cancer^[16–21]^; however, to date, no bibliometric analyses exist on GD. Therefore, we conducted a bibliometric and visual analysis of GD research published in the Web of Science Core Collection (WoSCC) database from 2003 to 2023. We aimed to summarize research achievements, unearth research hotspots, help researchers quickly and comprehensively understand the current research status, and provide directions for future studies.

## 2. Methods

### 2.1. Search strategy

**Figure 1.**
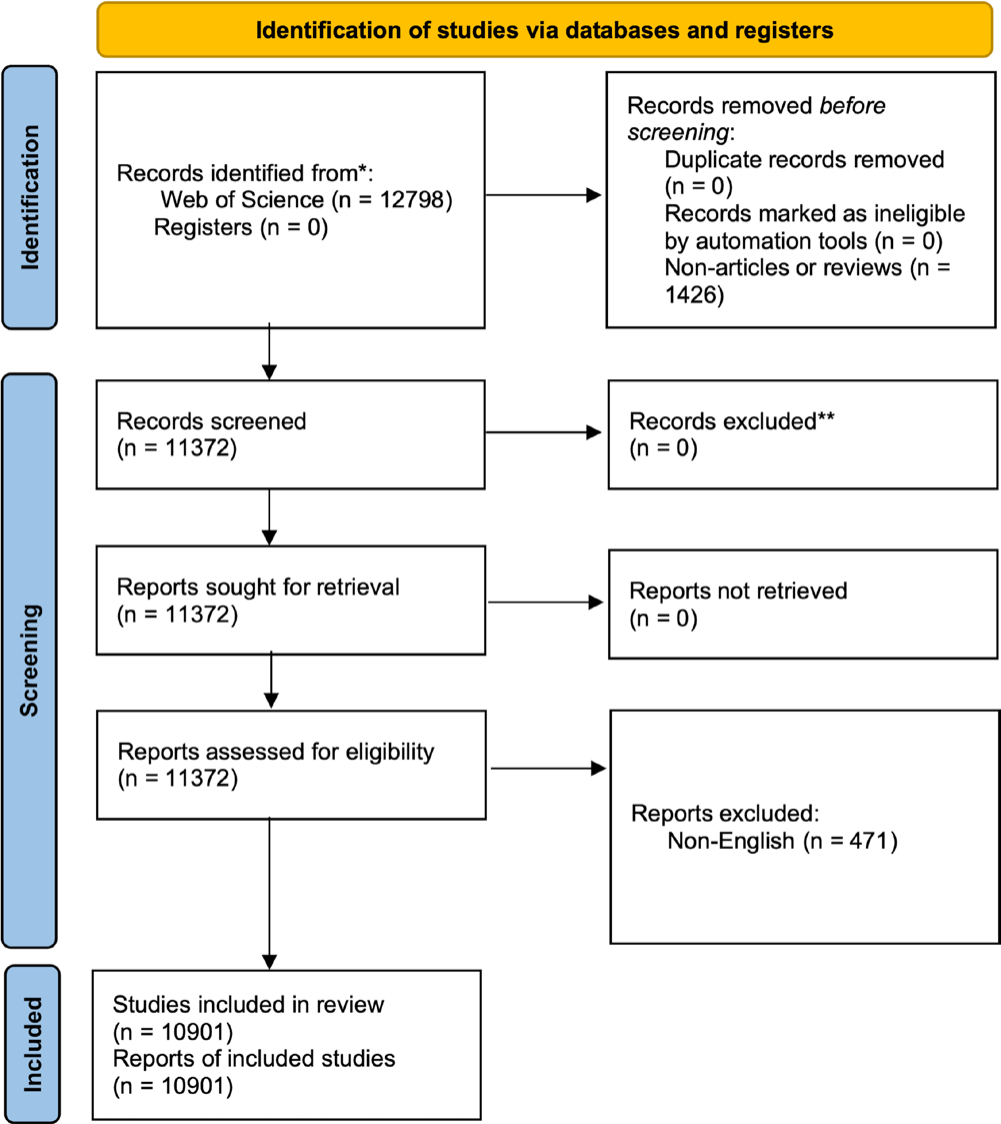
Flowchart of the publication screening process.

The WoS database, recognized as an authoritative source, served as the literature retrieval platform. We conducted a literature search on August 1, 2023, and downloaded all relevant documents. The search formula employed was TS = (Graves’ disease), covering the period from January 1, 2003 to July 31, 2023. The document types included articles and reviews, and the language was restricted to English, with no additional search constraints (see Figure 1).

### 2.2. Data analysis and visualization

In this study, the advanced version of CiteSpace and the R package Bibliometrix served as analysis tools to extract key information and integrate relevant data. We employed them to visualize analyses related to countries and institutions, journal and co-cited journal analyses, author and co-cited author analyses, co-cited literature analyses, and co-occurring keyword analyses. Additionally, we generated overlay maps for journals and employed citation burst analysis for the literature and keywords.

## 3. Results

### 3.1. Quantitative analysis of publications

**Figure 2.**
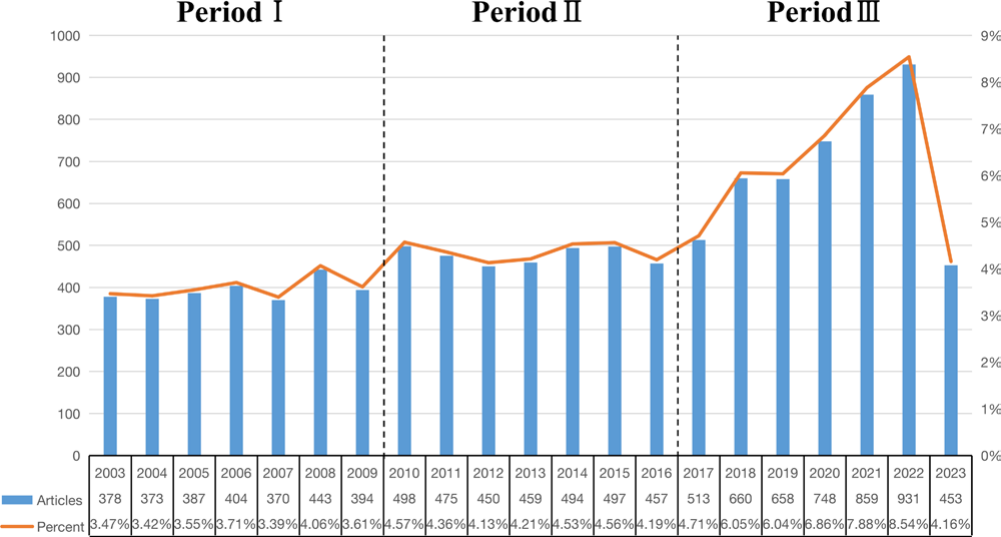
Annual output of publications.

The results of the literature analysis spanning the past 20 years revealed 12,798 studies related to GD, including 11,372 articles and reviews. Among these, 10,901 were English-language articles (see Fig. 1). The analysis of the annual publication volume (see Fig. 2) identified 3 distinct phases throughout the study period: Phase 1 (2003–2009), Phase 2 (2010–2016), and Phase 3 (2017–2023).

As depicted in Figure 2, Phase 1 contained relatively fewer studies on GD. In Phase 2, there was a slight increase in research quantity compared with Phase 1, although the annual publication rate remained relatively low. Notably, Phase 3 demonstrated a substantial increase in the number of publications compared with the preceding phases, indicating a generally rising trend. Importantly, the decrease in the annual publication count for 2023 was attributed to the inclusion of articles published until July 31st of that year.

### 3.2. Country analysis

The analyzed articles originated from 132 countries and regions, with the top 3 contributors being the US (n = 2377), China (n = 1315), and Italy (n = 916), accounting for 35.75% of all publications. The top 10 countries collectively contributed 64.69% of all publications (see Table 1).

**Table 1:** Top 10 countries and institutions in GD research.

Rank	Country	N (%)	Centrality	Institution	N (%)	Centrality
1	USA	2377 (18.44%)	0.21	University of California System	310 (2.41%)	0.5
2	China	1315 (10.20%)	0.04	University of Pisa	283 (2.20%)	0.33
3	Italy	916 (7.11%)	0.05	N8 Research Partnership	221 (1.72%)	0.18
4	Japan	886 (6.87%)	0.02	University of California Los Angeles	188 (1.46%)	0.03
5	England	690 (5.35%)	0.20	University of London	164 (1.28%)	0.12
6	Germany	622 (4.83%)	0.05	University of California Los Angeles Medical Center	152 (1.18%)	0.02
7	Poland	409 (3.17%)	0.02	UDICE-French Research Universities	151 (1.17%)	0.03
8	Turkey	392 (3.04%)	0.00	Harvard University	151 (1.17%)	0.35
9	South Korea	373 (2.89%)	0.05	Shanghai Jiao Tong University	150 (1.17%)	0.34
10	India	358 (2.78%)	0.04	David Geffen School of Medicine at UCLA	145 (1.13%)	0.02

**Figure 3.**
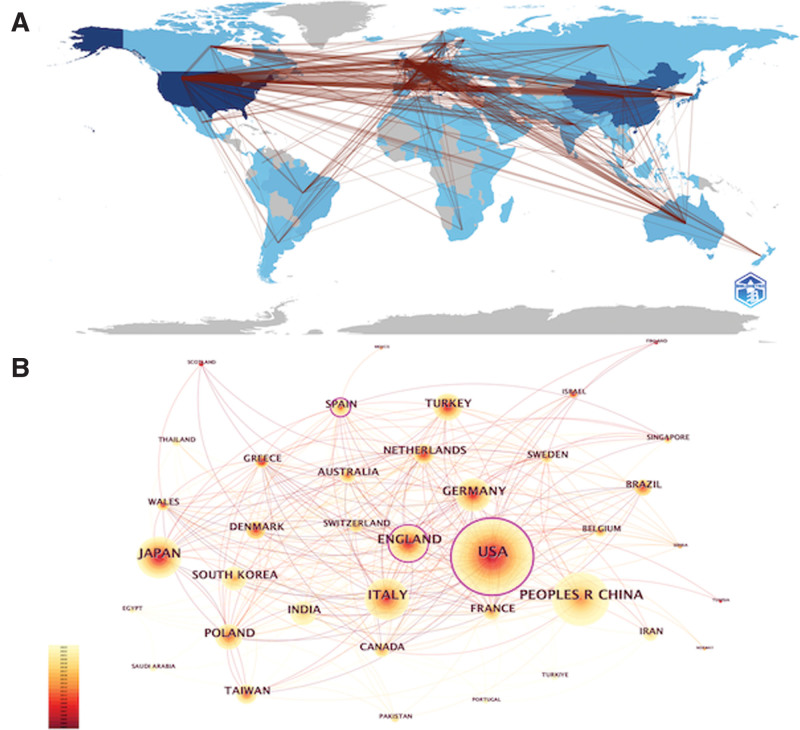
Geographic distribution (A) and visualization (B) of countries where GD research has been conducted.

To visualize collaboration patterns between countries, we constructed a cooperative network graph based on the publication volume and interactions of each country. Noteworthy collaborations include those between the US and China, the US and the UK, the UK and Germany, and Germany and Italy (see Fig. 3).

### 3.3. Institutional analysis

The corpus of literature for this study originated from 708 institutions, with the top 3 institutions being the University of California System (n = 310), the University of Pisa (n = 283), and the N8 Research Partnership (n = 221). Among the top ten institutions, 5 were from the US, 2 from the UK, one from France, one from Italy, and one from China (see Table 1).

**Figure 4.**
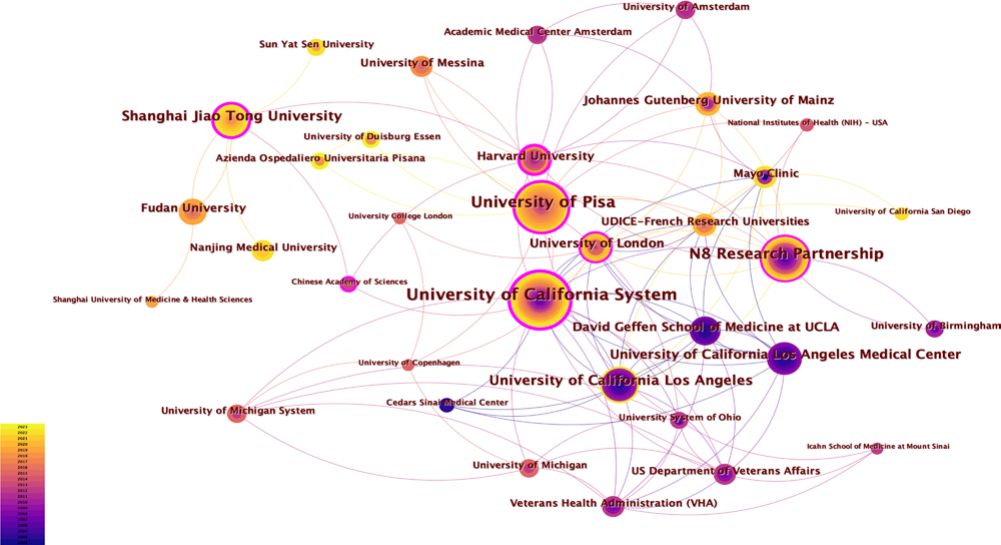
Visualization of institutions on GD research.

The collaborative networks between institutions revealed significant interactions. Notably, a close collaboration is observed among institutions within the University of California System, including the University of California, Los Angeles (UCLA); the University of California Los Angeles Medical Center; and the David Geffen School of Medicine at UCLA. Additionally, active cooperative relationships exist between the N8 Research Partnership and Harvard University as well as between the University of Pisa and the University System of Ohio (Fig. 4).

### 3.4. Analysis of journals and co-cited journals

The studied literature was distributed across 2402 journals. Table 2 presents the top 10 journals by publication volume, each featuring over 100 articles. The top 3 journals, *Thyroid, the Journal of Clinical Endocrinology and Metabolism*, and *Clinical Endocrinology*, have each published over 200 articles, totaling 1070 documents, which constitute 9.81% of the entire corpus. Among all journals, Thyroid boasts the highest output and impact factor (n = 469, 4.30%, IF = 6.6), followed by *the Journal of Clinical Endocrinology and Metabolism* (n = 375, 3.44%, IF = 5.8). The influence of journals is proportional to the number of co-citations. Thirty-two journals have been cited over 1000 times. Specifically, *the Journal of Clinical Endocrinology and Metabolism* has the highest number of citations (n = 6752), followed by *Thyroid* (n = 6218) and *Clinical Endocrinology* (n = 4935) (Table 3).

**Table 2:** Top 10 journals in GD research.

Rank	Journal	N (%)	IF (2022)	Quartile (2022)
1	Thyroid	469 (4.30%)	6.6	Q1
2	Journal Of Clinical Endocrinology Metabolism	375 (3.44%)	5.8	Q1
3	Clinical Endocrinology	226 (2.07%)	3.2	Q3
4	Journal Of Endocrinological Investigation	197 (1.81%)	5.467	Q2
5	Endocrine Journal	176 (1.61%)	2	Q4
6	European Journal Of Endocrinology	175 (1.61%)	5.8	Q1
7	Frontiers In Endocrinology	166 (1.52%)	5.2	Q1
8	Endocrine	144 (1.32%)	3.7	Q3
9	Ophthalmic Plastic And Reconstructive Surgery	126 (1.16%)	2	Q3
10	Plos One	122 (1.12%)	3.7	Q2

**Table 3:** Top 10 co-cited journals in GD research.

Rank	Co-cited journal	Co-citation (%)	Centrality	IF (2021)	Quartile (2021)
1	Journal Of Clinical Endocrinology Metabolism	6752 (5.85%)	0.37	5.8	Q1
2	Thyroid	6218 (5.39%)	0.2	6.6	Q1
3	Clinical Endocrinology	4935 (4.27%)	0.2	3.2	Q3
4	New England Journal Of Medicine	4696 (4.07%)	0.17	158.50	Q1
5	European Journal Of Endocrinology	4021 (3.48%)	0.24	5.80	Q1
6	Lancet	2940 (2.55%)	0.01	168.90	Q1
7	Journal Of Endocrinological Investigation	2710 (2.35%)	0.03	5.467	Q2
8	Endocrine Reviews	2422 (2.10%)	0.02	20.30	Q1
9	Journal Of Immunology	2034 (1.76%)	0.07	4.40	Q2
10	Journal Of Clinical Investigation	1809 (1.57%)	0.11	15.90	Q1

**Figure 5.**
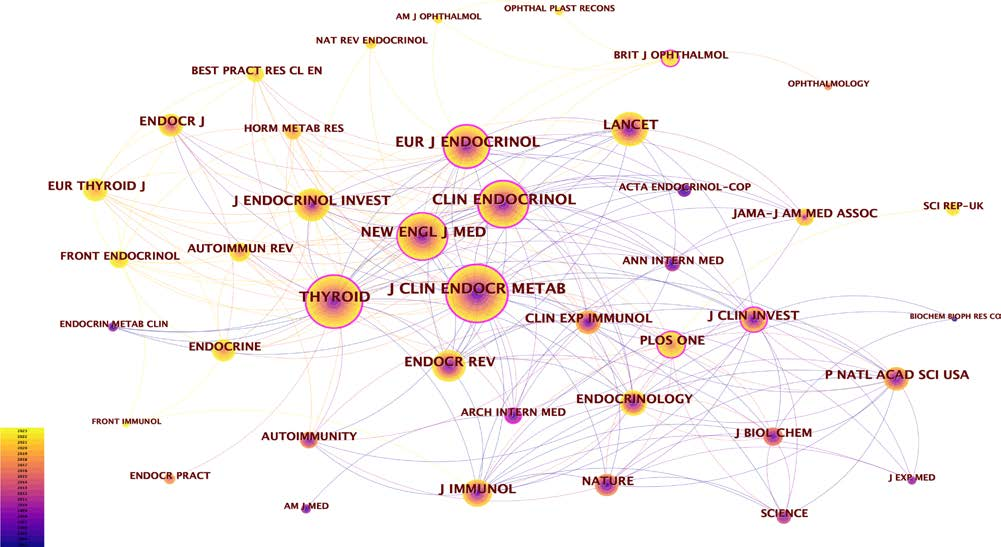
Visualization of co-cited journals on GD research.

**Figure 6.**
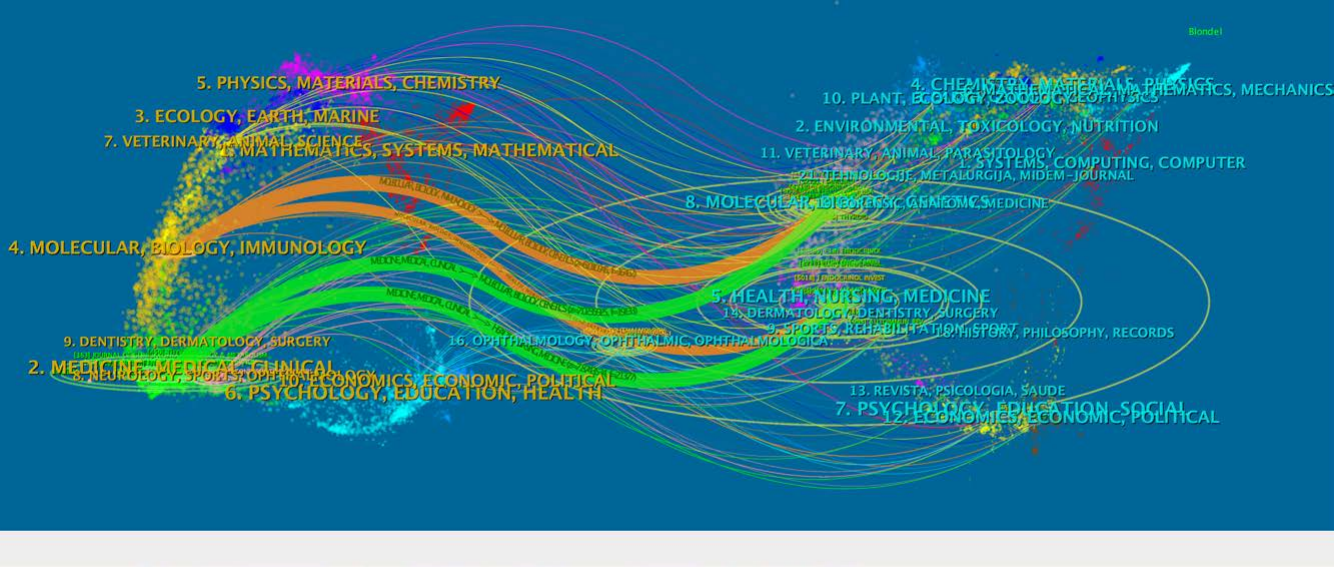
Dual-map overlay of journals on GD research.

Furthermore, co-citation analysis revealed robust citation relationships. *The Journal of Clinical Endocrinology and Metabolism* exhibited active citation ties with *Thyroid, New England Journal of Medicine*, and *Lancet* (see Fig. 5). The co-citation overlay map (see Fig. 6) elucidates the citation relationships, with the left side representing citing journals, the right side representing cited journals, and the lines indicating citation connections. The 4 thickest lines denote the main citation paths, suggesting that papers published in journals related to molecular/biology/immunology and medicine/medical/clinical fields were most frequently cited by articles in journals related to molecular/biology/genetics and health/nursing/medicine.

### 3.5. Analysis of authors and co-cited authors

**Figure 7.**
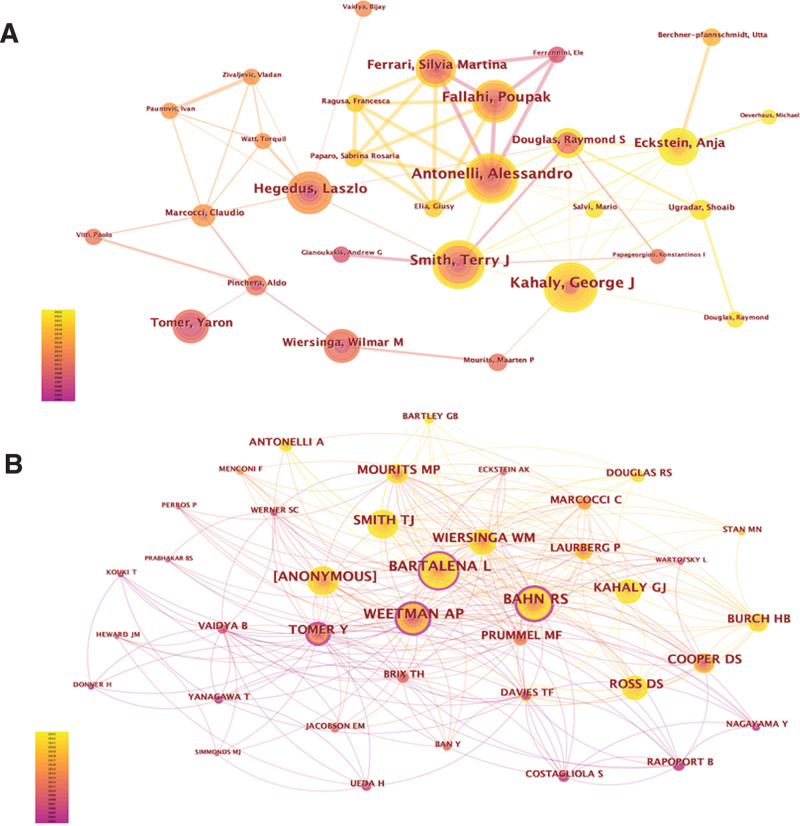
Visualization of authors (A) and co-cited authors (B) who have performed GD research.

A total of 2305 scholars have contributed to GD research, with Table 4 displaying the top 10 authors by publication volume. Antonelli Alessandro and Smith Terry shared the top position, with 80 publications each, followed by Kahaly George at 78 articles. Notably, all top 10 authors have published over 40 articles, and the top 5 authors have authored over 70 articles each. Figure 7 shows the collaborative relationships among several authors, such as Antonelli Alessandro with Smith Terry and Kahaly George with Douglas Raymond. Interestingly, Antonelli Alessandro collaborated closely with the top 5 authors.

**Table 4:** Top 10 authors and co-cited authors in GD research.

Rank	Author	Count (%)	Centrality	Co-cited author	Co-citation (%)	Centrality
1	Antonelli, Alessandro	80 (0.81%)	0.02	Bartalena L	1791 (3.95%)	0.17
2	Smith, Terry J	80 (0.81%)	0.04	Bahn Rs	1434 (3.16%)	0.2
3	Kahaly, George J	78 (0.79%)	0.07	Weetman Ap	1391 (3.07%)	0.37
4	Fallahi, Poupak	77 (0.78%)	0	Anonymous	1227 (2.71%)	0.03
5	Ferrari, Silvia Martina	74 (0.75%)	0	Smith Tj	1059 (2.34%)	0.04
6	Hegedus, Laszlo	60 (0.61%)	0.06	Kahaly Gj	904 (1.99%)	0.03
7	Douglas, Raymond S	51 (0.52%)	0.07	Wiersinga Wm	895 (1.97%)	0.08
8	Zhang, Jin-an	47 (0.48%)	0.02	Cooper Ds	784 (1.73%)	0.01
9	Tomer, Yaron	47 (0.48%)	0.01	Tomer Y	781 (1.72%)	0.22
10	Yoon, Jin Sook	45 (0.46%)	0.01	Mourits Mp	778 (1.72%)	0.04

Among the 436 co-cited authors, the top 10 have garnered over 700 citations each, and the top 5 authors have received over 1000 citations (see Table 4). Bartalena (n = 1791) emerges as the most frequently cited author, followed by Bahn (n = 1434) and Weetman (n = 1391). Notably, Smith Terry, Kahaly George, and Tomer Yaron are the only 3 scholars ranking in the top 10 for both publication volume and citation count.

### 3.6. Analysis of co-cited references

Over the past 2 decades, there have been 21,968 co-cited references in GD research. The top 10 co-cited references, each cited at least 100 times, are listed in Table 5. Notably, 4 of these articles originated from the New England Journal of Medicine and had the highest IF. Among all co-cited references, the paper by Ross et al, titled “2016 American Thyroid Association Guidelines for diagnosis and management of hyperthyroidism and other causes of thyrotoxicosis,” published in Thyroid, stands out as the most-cited (n = 408). Following closely is the article “The 2016 European Thyroid Association/European Group on Graves’ orbitopathy guidelines for the management of Graves’ orbitopathy” published in the European Thyroid Journal (n = 301). Notably, both studies were based on clinical guidelines.

**Table 5:** Top 10 co-cited references in GD research.

Rank	Author	Cited reference	Co-citation (%)	Centrality	Journal	IF (2021)
1	Ross DS (2016)^[22]^	2016 American Thyroid Association Guidelines for Diagnosis and Management of Hyperthyroidism and Other Causes of Thyrotoxicosis	408 (1.86%)	0.08	Thyroid	6.6
2	Bartalena L (2016)^[23]^	The 2016 European Thyroid Association/European Group on Graves’ Orbitopathy Guidelines for the Management of Graves’ Orbitopathy	301 (1.37%)	0.11	European Thyroid Journal	4.7
3	Smith TJ (2016)^[24]^	Graves’ Disease	296 (1.35%)	0.11	New England Journal of Medicine	158.50
4	Kahaly GJ (2018)^[25]^	2018 European Thyroid Association Guideline for the Management of Graves’ Hyperthyroidism	268 (1.22%)	0.04	European Thyroid Journal	4.7
5	Smith TJ (2017)^[26]^	Teprotumumab for Thyroid-Associated Ophthalmopathy	208 (0.95%)	0.25	New England Journal of Medicine	158.50
6	Bahn RS (2010)^[27]^	Graves’ ophthalmopathy	184 (0.84%)	0.22	New England Journal of Medicine	158.50
7	Douglas RS (2020)^[28]^	Teprotumumab for the Treatment of Active Thyroid Eye Disease	166 (0.76%)	0.09	New England Journal of Medicine	158.50
8	Bartalena L (2021)^[29]^	The 2021 European Group on Graves’ orbitopathy (EUGOGO) clinical practice guidelines for the medical management of Graves’ orbitopathy	147 (0.67%)	0.02	European Journal Of Endocrinology	5.8
9	Antonelli A (2015)^[30]^	Autoimmune thyroid disorders	143 (0.65%)	0.78	Autoimmunity Reviews	13.6
10	Ueda H (2003)^[31]^	Association of the T-cell regulatory gene CTLA-4 with susceptibility to autoimmune disease	136 (0.62%)	0.25	Nature	64.80

**Figure 8.**
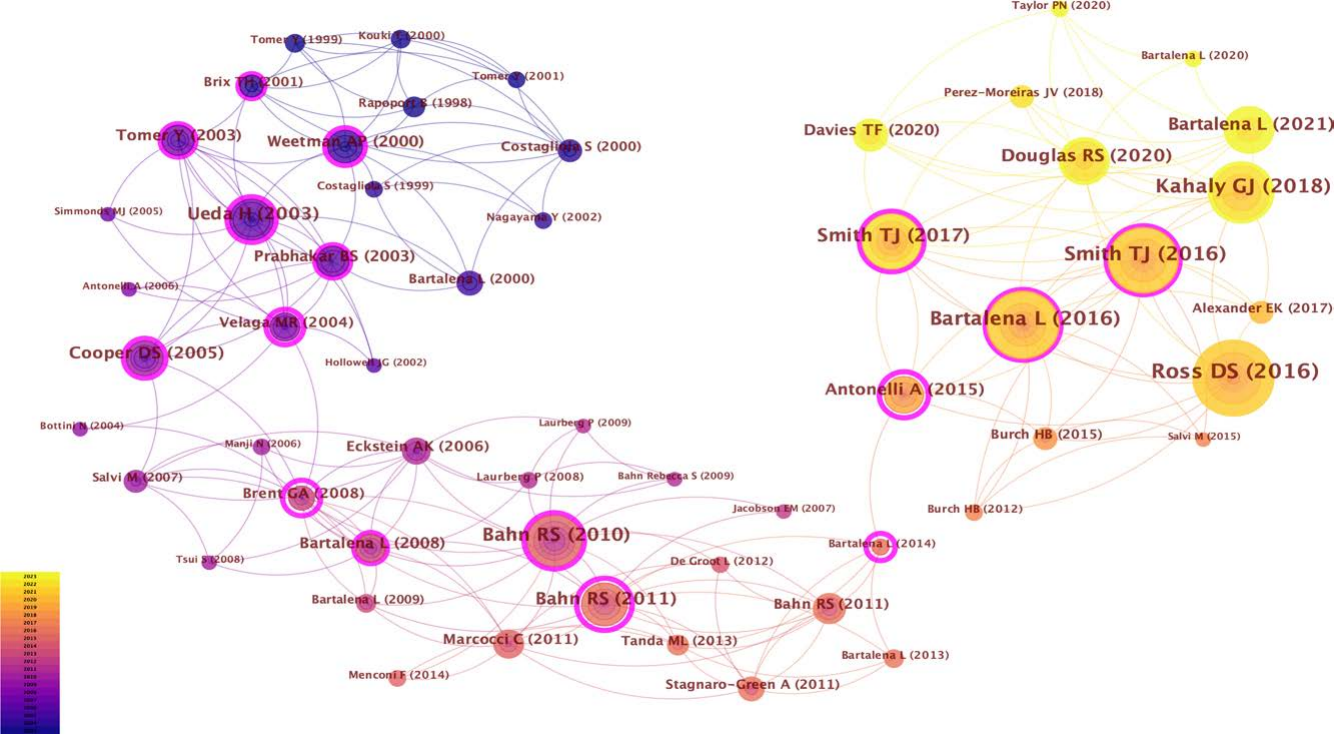
Visualization of co-cited references on GD research.

Additionally, visual analysis of co-cited references revealed active citation relationships among the top 10 co-cited references, except for “Graves’ ophthalmopathy” by Bahn et al in 2010 and “Association of the T-cell regulatory gene cytotoxic T lymphocyte antigen-4 (CTLA-4) with susceptibility to autoimmune disease” by Ueda et al in 2003 (see Fig. 8).

### 3.7. References with citation bursts

**Figure 9.**
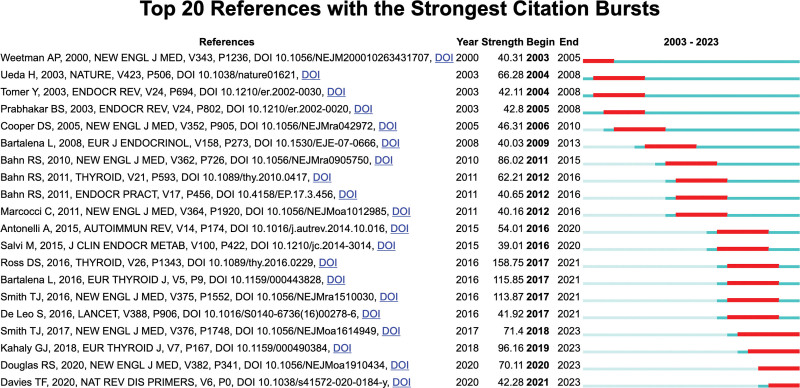
Top 20 references with strong citation bursts.

“Bursting references” denote literature that experiences a high-frequency of citations within a specific field during a particular period. Figure 9 shows the top 20 bursting references. The blue line segments represent time intervals, whereas the red line segments indicate periods of frequent citations. Figure 9 indicates that bursting references emerged each year. The burst strength of these 20 references ranged from 39.01 to 158.75, with durations of endurance spanning 2 to 5 years.

Among these references, the top 3 in burst strength are the “2016 American Thyroid Association guidelines for diagnosis and management of hyperthyroidism and other causes of thyrotoxicosis” (strength = 158.75), the “2016 European Thyroid Association/European Group on Graves’ orbitopathy guidelines for the management of Graves’ orbitopathy” (strength = 115.85), and “Graves’ disease” (strength = 113.87). Notably, these references not only exhibited the highest burst strength but also endured for a period of 4 or more years.

### 3.8. Keyword analysis

A network graph of keywords revealed research hotspots within a specific field. Table 6 presents the top 40 high-frequency keywords used in GD research. “Graves’ disease,” “autoimmune thyroid disease,” and “Graves ophthalmopathy” claim the top 3 spots, each appearing over 2000 times, while “hyperthyroidism” and “management” exceed 1000 occurrences. The top 15 keywords surpassed 700 occurrences, and the top 40 keywords appeared at least 190 times, representing the primary research direction for GD.

**Table 6:** Top 10 keywords in GD research.

Rank	Keywords	Count	Centrality	Rank	Keywords	Count	Centrality
1	graves disease	4917	0.03	21	regulatory t cells	441	0.04
2	autoimmune thyroid disease	2664	0.03	22	antithyroid drugs	415	0.02
3	graves ophthalmopathy	2167	0.12	23	thyroid function	403	0.02
4	hyperthyroidism	1878	0.03	24	rheumatoid arthritis	383	0.04
5	management	1339	0.05	25	hypothyroidism	366	0.02
6	thyrotropin receptor	959	0.05	26	surgery	355	0.02
7	gene	804	0.05	27	follow up	337	0.02
8	expression	799	0.03	28	cells	330	0.03
9	prevalence	796	0.05	29	dysfunction	324	0.02
10	association	785	0.04	30	tsh	297	0.02
11	autoantibody	761	0.04	31	children	288	0.02
12	hashimotos thyroiditis	752	0.04	32	orbital fibroblasts	280	0.04
13	risk factors	742	0.03	33	systemic lupus erythematosus	276	0.02
14	pathogenesis	714	0.03	34	guidelines	268	0.01
15	therapy	711	0.04	35	binding	233	0.06
16	population	647	0.03	36	quality of life	231	0.02
17	diagnosis	617	0.03	37	nodules	222	0.02
18	thyroid cancer	595	0.03	38	classification	220	0.03
19	radioiodine therapy	563	0.02	39	methimazole	210	0.02
20	susceptibility	477	0.06	40	multiple sclerosis	195	0.02

**Figure 10.**
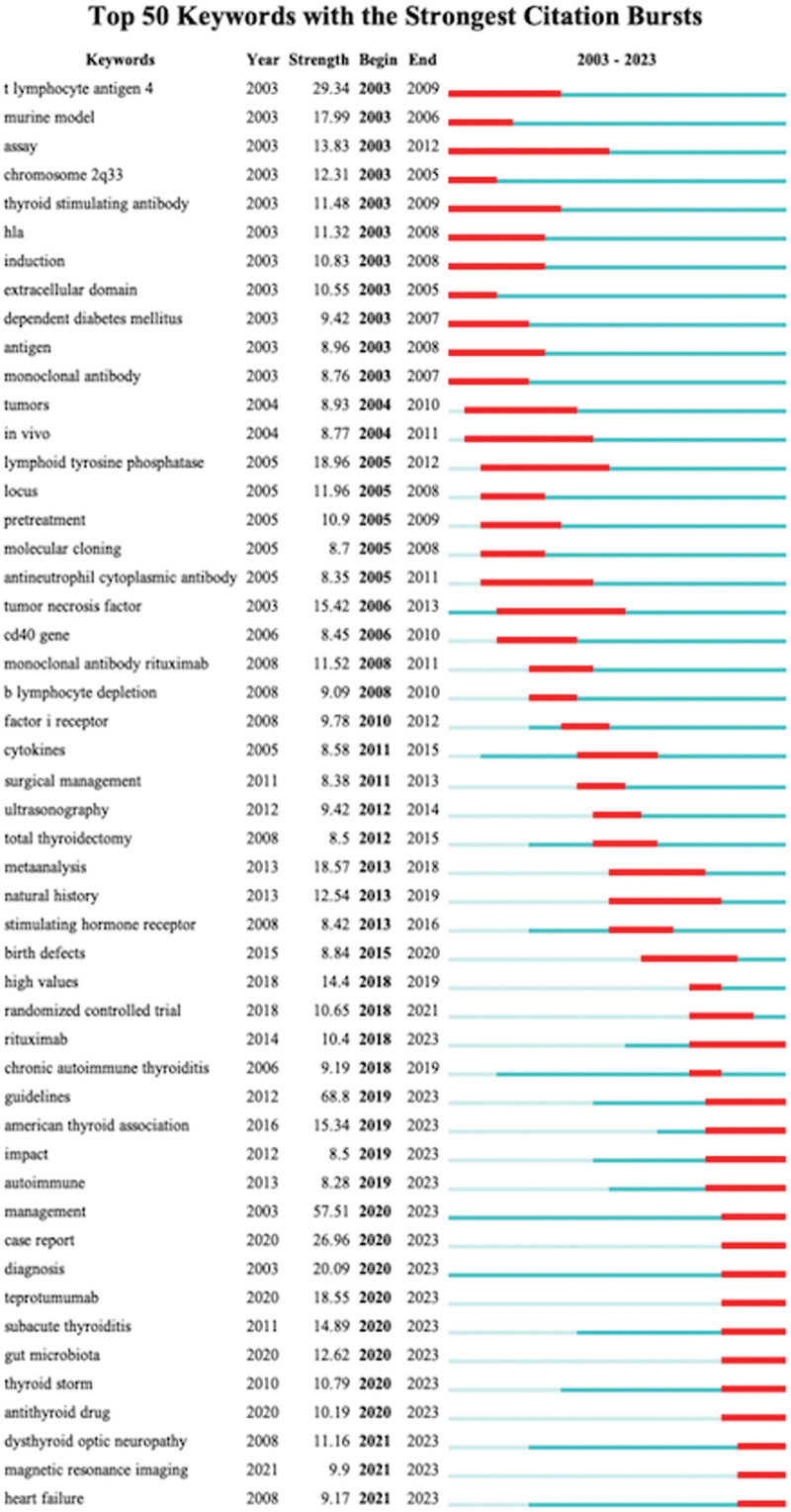
Top 50 keywords with strong citation bursts.

**Figure 11.**
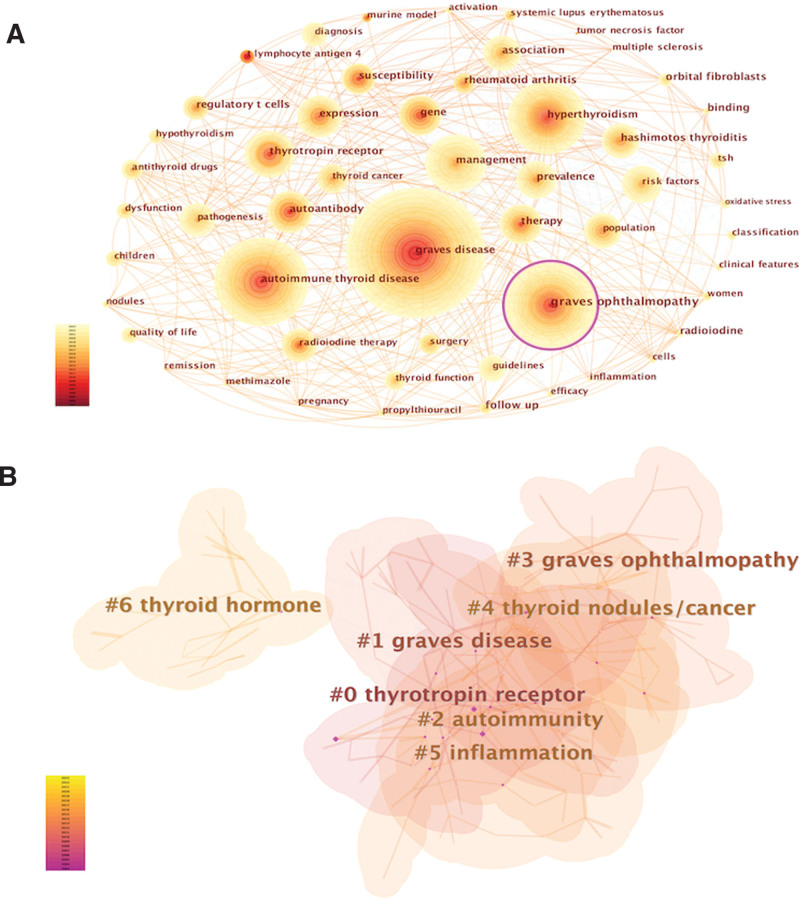
Visualization of the analysis of keywords (A) and keyword clusters (B) on GD research.

Figure 10 illustrates keywords with strong burst citations. We selected the top 50 keywords with burst citations extending to the year 2023. Among them, “guidelines” (strength = 68.8), “management” (strength = 57.51), “T lymphocyte antigen-4” (strength = 29.34), “case report” (strength = 26.96), and “diagnosis” (strength = 20.09) take up the top 5 spots in burst strength. Additionally, we conducted a cluster analysis on keywords and obtained 7 clusters (Fig. 11).

## 4. Discussion

### 4.1. General information

Medical journals have played a pivotal role in the historical dissemination of medical information worldwide, spanning over 3 centuries from the inception of the first traditional journals to today’ s open-access publications.^[32]^ The proliferation of journals and the increasing volume of published articles reflect the continuous progress and evolution of medical research. However, the advent of the information explosion has also posed challenges in obtaining a comprehensive understanding of knowledge in specific fields, during a given period, and across particular topics.^[33]^

The emergence of bibliometric theories and tools offers effective solutions to these challenges.^[34]^ As a research methodology, bibliometrics enables the systematic and quantitative analysis of scientific literature, providing valuable insights into the landscape of scholarly communication. By leveraging bibliometric approaches, researchers can navigate the vast sea of published literature, identify trends, and extract meaningful patterns in scientific research.

The history of GD dates back to 1835 when Irish physician Robert James Graves first reported this condition, marking nearly 2 centuries of research.^[35,36]^ In this study, we conducted a systematic search of the WoS database spanning the past 2 decades (2003–2023) to compile a comprehensive overview of GD research. After applying meticulous exclusion criteria, we included 10,901 English-language papers from 708 institutions across 132 countries, involving 2305 scholars. These papers were published in 2402 different journals and referenced in 21,968 articles. The analysis revealed a noticeable increase in publications since 2017, suggesting a growing focus on GD research in recent years. Collaboration appears to be a key trend, with multi-institutional and international cooperation emerging as a direction for future research. Although current collaborations primarily involve a few countries, institutions, and domestic authors, fostering broader partnerships is imperative for long-term development.^[37]^ In examining publication trends and citation patterns, we observed that most GD research was published in endocrinology-related journals. However, noteworthy contributions from high-impact multidisciplinary journals underscore the significance and broad relevance of GD research. Dual-map overlay analysis further implies that research on GD straddles both the basic and clinical domains, reflecting a phase of concurrent development in these areas. Nevertheless, there is a substantial difference in the progress achieved by basic and clinical research, with clinical studies, especially guidelines and management-related publications, dominating the landscape. Among the top 10 most-cited articles, 4 are clinical management guidelines, 2 focus on clinical pharmacology for GD ophthalmopathy, 3 are reviews related to GD, and only one represents fundamental research.^[22–30]^ This study, conducted by Ueda et al, identified a close association of the T-cell regulatory gene CTLA-4 with GD pathogenesis through comparative gene analysis in different participants and splice studies in mice published in Nature.^[31]^ A noteworthy observation emerged from the analysis of the authors and co-cited authors. The top 10 authors, all with 40 or more publications, included Smith Terry, who contributed 2 highly co-cited articles published in the New England Journal of Medicine. A PubMed search indicated that many of these authors had consistently published studies over the past 5 years of the study period. Hence, these prolific researchers warrant attention as their work may represent potential paths for future exploration, providing valuable insights and inspiration.

### 4.2. Hotspots and frontiers

The eruption of cited references and the analysis of keywords serve as valuable tools for discerning emerging themes across various periods of GD research, facilitating the rapid capture of evolving hotspots and their trajectories. Following a meticulous examination of the top 20 burst references and clustering analysis of the foremost 50 keywords, our deductions converged on several pivotal domains characterizing GD investigations, including clinical management (diagnosis and treatment) and mechanisms (autoimmunity and inflammation) of GD, Graves’ Ophthalmopathy, and Thyroid Nodules and Thyroid Cancer in GD.

#### 4.2.1. Clinical management of Graves’ disease

##### 4.2.1.1. Diagnosis

The diagnostic protocol for GD is well-established. First, suspected patients should exhibit specific signs and symptoms of hyperthyroidism, such as sweating, palpitations, tremors, behavioral changes, a lack of concentration, emotional instability, accelerated growth and bone maturation, thyroid enlargement, pretibial myxedema, and infiltrative exophthalmos (Graves’ ophthalmopathy).^[3]^ Second, biochemical blood tests should indicate suppressed thyroid-stimulating hormone (TSH), elevated free triiodothyronine, and/or elevated free thyroxine concentrations (an elevated elevated free triiodothyronine level is a more sensitive marker of GD than an elevated and/or elevated free thyroxine level).^[38]^ Third, immunological blood tests should reveal elevated titers of TSH receptor antibodies (TRAb). The diagnosis of GD is confirmed when all 3 criteria are met.^[22]^ For patients with moderately elevated TRAb, a diagnosis can be established if radioactive iodine uptake testing indicates diffuse uptake and an increased uptake rate.^[24]^ The diagnostic process for GD in children mirrors that in adults. However, owing to its rarity and often subtle symptoms, this condition is frequently misattributed to primary psychological conditions or gastrointestinal and cardiovascular disorders, leading to potential harm to developing children and adolescents.^[39]^ Notably, pregnancy itself may cause palpitations and sweating, and increased estrogen secretion can elevate total serum thyroxine levels.^[40]^ Since pregnant women cannot undergo radioactive testing, diagnosing GD during pregnancy is extremely challenging when the patient tests negative for TSH receptor antibodies.^[41]^

##### 4.2.1.2. Treatment

Although treatment approaches for GD vary among countries and regions, it is generally agreed that patients should undergo one of the following treatment methods: antithyroid drug therapy, radioactive iodine therapy, or thyroidectomy.^[22]^

###### 4.2.1.2.1. Antithyroid drug therapy

Currently, in Europe, Latin America, and Japan, antithyroid drug therapy is the primary treatment option^[22,25]^; although radioactive iodine therapy is favored by physicians in the US, the use of antithyroid drugs is gradually increasing, indicating a trend toward becoming a primary treatment approach.^[24,42]^ Although antithyroid drugs do not cure GD, a sufficient and prolonged course of treatment is highly effective in controlling hyperthyroidism.^[22]^ Additionally, antithyroid drug therapy is the preferred option for GD in children, adolescents, and pregnant women.^[43]^ The choice of antithyroid drugs includes propylthiouracil during the first trimester of pregnancy, thyroid storms, and adverse reactions to other antithyroid drugs. Methimazole is recommended for all other patients, including children and adolescents.^[22,43]^ The typical treatment duration with methimazole is 12 to 18 months, with discontinuation when the TRAb and TSH levels normalize. However, if hyperthyroidism persists after completion of the treatment regimen, options include continued low-dose methimazole, radioactive iodine therapy, or surgical intervention.^[22]^

###### 4.2.1.2.2. Radioactive iodine therapy

Radioactive iodine is generally considered the optimal treatment for patients with mild thyroid enlargement or those for whom antithyroid drug therapy is ineffective or contraindicated.^[22]^ Typically, a single dose of adequate radioactive iodine is administered to induce thyroid hypofunction in patients with GD. In children, a reduced dose of radioactive iodine is used to achieve a fairly rapid reduction in thyroid function and minimize the risk of recurrence.^[22,44]^ Thyroid function should be monitored continuously for 6 months after radioactive iodine therapy until the patient’ s thyroid function stabilizes through thyroid hormone replacement therapy.^[22]^ Radioactive iodine is not recommended for patients with Graves’ ophthalmopathy owing to the inherent risk of exacerbating the condition. Smoking individuals are also discouraged from radioactive iodine therapy, as they are more prone to Graves’ ophthalmopathy.^[45,46]^ Furthermore, studies suggest a long-term increase in cancer risk in patients receiving radioactive iodine; hence, this viewpoint remains controversial.^[47,48]^

###### 4.2.1.2.3. Surgical treatment: thyroidectomy

Thyroidectomy is the preferred treatment for symptomatic compression or large goiters (≥ 80 grams) in patients with moderate to severe active Graves’ ophthalmopathy.^[22]^ Better results and lower postoperative morbidity rates were associated with surgeons who had more experience and higher surgical volumes.^[49]^ Although the mortality rate of thyroidectomy is extremely low (<0.1%), complications such as postoperative hypocalcemia warrant attention.^[50]^

For the clinical management of GD, addressing the challenge of a rational, early, and accurate diagnosis in children and pregnant women is imperative and represents a potential avenue for future research. Additionally, further research should focus on the dosage, treatment duration, and cessation criteria for antithyroid drug therapy; the potential long-term risks of radioactive iodine therapy for cancer incidence; and predictive and preventive measures for postoperative hypocalcemia following surgical treatment.

###### 4.2.1.2.4. Thyroid-stimulating hormone receptor and thyroid hormones

As mentioned earlier, GD is characterized by the abnormal increase in TRAb, leading to enhanced synthesis and secretion of thyroid hormones.^[51]^ Three types of TRAb have been identified: thyroid-stimulating antibodies, thyroid-blocking antibodies, and neutral antibodies.^[7,8]^ GD is closely associated with thyroid-stimulating antibodies.^[7]^ Duan et al^[52]^ examined the molecular structures of TSHs, TSH receptors, human-activated antibodies, and inhibitory antibodies. They identified the key amino acid sites crucial for hormone receptor-specific recognition. By comparing the states of activated antibodies, inhibitory antibodies, and the binding of TSHs to TSH receptors, activated antibodies were found to induce an upright active conformation of the extracellular domain (ECD) of TSH receptor (TSHR), causing receptor-similar conformational changes and subsequent activation.^[52]^ By contrast, inhibitory antibodies competitively bind to hormone receptors and inhibit receptor activation without inducing receptor-similar conformational changes.^[52]^ This molecular-level study provides the first explanation of the pathogenic mechanism of GD and lays the foundation for the development of novel therapeutic drugs. Further research by Faust shed light on the aforementioned pathogenic mechanism, proposed a model for the physiological and pathological activation of TSHR, and suggested the potential extension of this mechanism to other G protein-coupled receptors with large ECDs.^[53]^ Duan et al^[54]^ designed a novel therapy using chimeric antigen receptor T cells. These cells demonstrate the ability to recognize and effectively eliminate B lymphocytes producing TRAb both in vitro and in vivo, offering a promising and innovative immunotherapy for GD.^[54]^

On the clinical front, given the critical importance of TSH receptor antibodies for diagnosis and treatment, there is a major research focus on developing more sensitive detection methods and effectively reducing TSH receptor levels. Currently, 3 common methods are used to detect TSH receptor antibodies: competitive immunoassays, bioassays, and enzyme-linked immunosorbent assays (ELISA).^[55]^ Competitive immunoassays do not distinguish between stimulating and blocking active antibodies but allow for quantification of the binding capacity of TSH receptor antibodies.^[56,57]^ Bioassays measure the stimulatory or inhibitory effects of TRAb by detecting cAMP production and intracellular TSHR signals.^[57,58]^ ELISA, which is more commonly used in research than in clinical settings, is based on the inhibition of human monoclonal TRAb (M22) binding.^[59]^ Several studies have compared instruments from different manufacturers for TSH receptor antibody detection, aiming to identify faster, more sensitive, accurate, and cost-effective detection methods.^[56,60,61]^

##### 4.2.1.3. Autoimmunity and inflammation

GD, an organ-specific autoimmune disorder, has been studied continuously, revealing novel findings related to its autoimmune response and inflammatory processes.^[1,2]^ Janyga et al^[62]^ identified a significant elevation in IL-23 and IL-31 levels in GD patients by measuring 15 cytokines associated with Th17 and Treg lymphocytes in the serum of different populations. IL-23, a key cytokine controlling peripheral tissue inflammation, is positively correlated with the severity of autoimmune disease symptoms, along with IL-31 and IL-33 levels.^[62,63]^ When IL-23 is activated, certain lymphocytes, including T cells, secrete cytokines such as IL-17, IL-22, TNF-α, and IFN-γ, enhancing the immune response.^[64]^ IL-23 induces the formation of pathogenic Th17 cells and inhibits the reactivity of Treg cells by suppressing the responsiveness of IL-33 and reducing the differentiation of Treg cells.^[65,66]^ This indicates a crucial role for IL-23 and IL-31 in GD pathogenesis.

Another study suggests that deficiencies in regulatory T cells (Treg) and regulatory B cells, imbalances between Treg and Th17 lymphocytes, and abnormal production of anti-inflammatory cytokines significantly impact the progression of autoimmune thyroid diseases, including GD.^[67]^ This also implies that deficiencies in minerals such as selenium, zinc, iron, copper, calcium, and magnesium may promote oxidative stress, affect immune function, and further contribute to GD. Supplementation with these minerals has been proposed as an immunomodulatory approach for the treatment of GD.^[67]^

Further research indicates that an imbalance between Treg and Th17 cells may lead to the development of GD. Various factors associated with the differentiation of Treg and Th17 cells have been identified in various pathological states. For example, low levels of nuclear receptor 4 group A in pathological states reduce the differentiation of Treg cells, whereas abnormal overexpression of IL-2 and C-C chemokine receptor type 6 induces the differentiation of Th17 cells and promotes their migration to the thyroid.^[68–70]^ Although earlier studies have attributed the occurrence of GD to an imbalance between helper T (Th) 1 and 2 cells, recent research on the balance between Th17 and Treg cells has introduced a new perspective.^[71]^ Investigating the differentiation of various T-cell subsets in GD (especially Th17 and Treg cells) to identify potential therapeutic targets is a promising current research focus.

##### 4.2.1.4. Graves’ ophthalmopathy

Graves’ ophthalmopathy is an autoimmune disease affecting the tissues behind the eyes. It predominantly occurs in patients with hyperthyroidism, with approximately 30% of cases being associated with GD. However, it is occasionally observed in patients with normal thyroid function or even hypothyroidism due to chronic thyroiditis.^[27,29]^ The exact mechanism underlying this condition is not fully understood; however, TSH receptor antibodies are widely recognized as significant contributors.^[72]^

Research suggests that fibroblasts initiate 2 key pathways under the stimulation of TSH receptor antibodies and T-cell activation.^[73]^ First, they respond to stimuli by producing glycosaminoglycans (GAGs), of which hyaluronic acid is the most prominent component. The accumulation of these hydrophilic molecules leads to the swelling of the extraocular muscles. Second, these cells further increase the volume of the extraocular muscles by promoting fat generation. Eyeball protrusion and other clinical manifestations of Graves’ orbitopathy result from these processes. In addition, it has been hypothesized that the IGF-1 receptor present in fibroblasts synergistically interacts with TSH receptor antibodies to promote the production of GAGs. Although this reaction provides some insight into the pathogenic mechanism of Graves’ ophthalmopathy, it is neither detailed nor comprehensive.

Currently, research on Graves’ ophthalmopathy primarily focuses on its clinical management. The European Group on Graves Orbitopathy released clinical practice guidelines in 2016 and 2021, ranking among the top 10 most-cited studies in the past 2 decades. The latest guidelines provide evidence-based treatment recommendations for Graves’ ophthalmopathy at different levels of severity and incorporate clinical management during viral pandemics.^[29]^ Recent research on Graves’ ophthalmopathy has emphasized clinical management and the inadequacies of basic research. The cyclic process of identifying clinical issues, addressing them through scientific research, and applying the results to clinical practice constitutes a beneficial feedback loop between clinical and basic research. This approach holds promise as a potential research method for Graves’ ophthalmopathy.

##### 4.2.1.5. Thyroid nodules and thyroid cancer in Graves’ disease

In recent years, an increasing number of institutions have published case reports on the association between GD, thyroid nodules, and thyroid cancer, indicating a growing scholarly interest in their correlation. A study involving 5025 patients with GD and 20,100 frequency-matched patients without GD demonstrated a higher risk of cancer among patients with GD. In particular, the risks of developing breast and thyroid cancers within 3 and 6 years were elevated, prompting the proposal of preventive strategies for thyroid and breast cancers.^[74]^ Another study suggested that the occurrence of thyroid nodules and the risk of thyroid cancer in patients with GD might increase with age.^[75]^ These conclusions are consistent with those of other studies.^[76,77]^ However, the mechanisms underlying the development of thyroid nodules and cancer in patients with GD remain unclear. Some studies have proposed a crucial role of thyroid-stimulating immunoglobulin in this process. However, there is an ongoing debate regarding whether TRAb levels positively or negatively correlate with the incidence of thyroid cancer in patients with GD.^[75,77,78]^ Future research should provide evidence to substantiate this correlation and unveil the underlying mechanisms to achieve early prediction, diagnosis, and treatment.

##### 4.2.1.6. Advantages and shortcomings

This study has several unique advantages over traditional reviews. First, we conducted a systematic analysis of GD using a bibliometric methodology, providing comprehensive guidance for researchers in this field. Second, our use of bibliometric tools such as CiteSpace and the R package Bibliometrix ensures a more objective data analysis. Finally, our results showcase trends and hotspots in GD research. However, this study has some limitations. First, we sourced our research data solely from the WoSCC database, which may have led to a reduction in the number of literature items that met the criteria. Second, the cutoff date for the publications included in our analysis was July 31, 2023; thus, our study may not entirely reflect the actual situation in 2023. Finally, we only included studies published in English, which might imply the omission of literature in other languages.

## 5. Conclusion

Our study represents the inaugural application of CiteSpace and the R package Bibliometrix, which enabled us to conduct a bibliometric analysis of the progress and trends in GD research. We comprehensively summarized the global research outcomes of GD from 2003 to 2023. The findings indicate a gradual increase in publications over the past 5 years, highlighting the growing trend of collaboration among various countries, institutions, and scholars. Current research focuses primarily on clinical management, TSH receptors and thyroid hormones, autoimmunity and inflammation, Graves’ ophthalmopathy, thyroid nodules, and thyroid cancer. We hope that this study serves as a valuable resource for fellow scholars, offering insights into the current state of GD research and aiding in the identification of prospective research directions.

## Acknowledgments

This is a short text to acknowledge the contributions of specific colleagues, institutions, or agencies that aided the efforts of the authors.

## Author contributions

**Conceptualization:** Yan Yang.

**Data curation:** Yan Yang, Peijin Li.

**Formal analysis:** Qian Wang.

**Funding acquisition:** Zhiguo Ding.

**Methodology:** Yan Yang, Peijin Li, Tao Liu, Qian Wang.

**Supervision:** Zhiguo Ding.

**Validation:** Peijin Li, Tao Liu, Qian Wang.

**Visualization:** Tao Liu.

**Writing – original draft:** Yan Yang, Peijin Li.

**Writing – review & editing:** Chunjian Zhou, Feng Liu, Tao Liu, Qian Wang, Zhiguo Ding.
